# A Low-Cost Sensor Network for Monitoring Peatland

**DOI:** 10.3390/s24186019

**Published:** 2024-09-18

**Authors:** Hazel Louise Mitchell, Simon J. Cox, Hugh G. Lewis

**Affiliations:** Computational Engineering and Design Group, Faculty of Engineering and Physical Sciences, University of Southampton, Southampton SO17 1BJ, UK; s.j.cox@soton.ac.uk (S.J.C.); h.g.lewis@soton.ac.uk (H.G.L.)

**Keywords:** peat, sensor network, IoT

## Abstract

Peatlands across the world are vital carbon stores. However, human activities have caused the degradation of many sites, increasing their greenhouse gas emissions and vulnerability to wildfires. Comprehensive monitoring of peatlands is essential for their protection, tracking degradation and restoration, but current techniques are limited by cost, poor reliability and low spatial or temporal resolution. This paper covers the research, development, deployment and performance of a resilient and modular multi-purpose wireless sensor network as an alternative means of monitoring peatlands. The sensor network consists of four sensor nodes and a gateway and measures temperature, humidity, soil moisture, carbon dioxide and methane. The sensor nodes transmit measured data over LoRaWAN to The Things Network every 30 min. To increase the maximum possible deployment duration, a novel datastring encoder was implemented which reduced the transmitted datastring length by 23%. This system was deployed in a New Forest (Hampshire, UK) peatland site for two months and collected more than 7500 measurements. This deployment demonstrated that low-cost sensor networks have the potential to improve the temporal and spatial resolution of peatland emission monitoring beyond what is achievable with traditional monitoring techniques.

## 1. Introduction

UK peatlands are estimated to hold around 5.5 billion tonnes of sequestered car-bon—many times the UK’s annual greenhouse gas (GHG) emissions [1]. Healthy peatlands take in carbon dioxide from the atmosphere and release far smaller quantities of methane [2], steadily trapping organic carbon in the soil [1]. However, damage and mismanagement of peatlands can affect the balance of carbon absorption and release, increasing the overall greenhouse gas emissions and the climate warming potential of peatlands [3,4].

The main greenhouse gases identified in emissions from peatlands are carbon dioxide (CO_2_), methane (CH_4_), nitrous oxide (N_2_O), volatile organic compounds (VOCs) and nitrogen (N) [5,6]. At present, most studies focus on carbon dioxide and methane emissions [7,8].

Manual data collection has formed the basis of most historical studies and remains a useful means of gathering initial site data. Associated techniques include flux chambers, coring, dipwells and visual surveys [6,9]. Typically, manual techniques are better suited to the general health assessment of peatlands, rather than investigating the immediate effects of extreme weather events or wildfires. Some limitations of manual techniques include disturbance to the peat and local wildlife, high costs of transportation and staff hours, and safety risks. Where site access is limited by flooding or fires, manual data collection may even become impossible, further limiting the studies that can be performed.

Conversely, remote sensing, e.g., by satellite or aircraft, can be used to acquire over much larger areas than manual techniques, without being affected by surface conditions [10,11,12,13]. However, the spatial resolution of satellites is limited [12], and aerial surveys require trained pilots or expensive unmanned aircraft, permits, and favourable flying conditions [11]. Furthermore, sites obscured by foliage, snow, or water may be impossible to survey remotely [11,13].

The Internet of Things (IoT) has become an increasingly prominent topic both as a topic of research and in our daily lives. Fundamentally, IoT is the concept of distributed, connected electronic sensors and devices working in unison despite physical separation [14]. In doing so, tasks that would be impossible for a single device can be accomplished, such as Smart City management [15,16,17] or monitoring air pollution across broad urban areas [18,19].

IoT networks are typically implemented in several layers: Things, Gateways, Edge Computing and the Cloud [20,21]. At the most physical layer, Things, or “nodes”, record measurements or interact with their environment. As sensor nodes are often small low-power devices, the long-range transmission of data payloads is handled by the next layer: gateways. These are devices that handle communication in a local area, much like WiFi routers. This system architecture offers a number of benefits in the context of collecting data in remote environments, such as peatlands. Human presence at the deployment site is only required during deployment, repair visits, or retrieval of the system; data are collected and transmitted autonomously, and improved temporal and spatial resolution can be achieved without increasing labour.

The outcomes of peatland management strategies, such as rewetting and afforestation, remain unpredictable and require long-term (multi-year) monitoring [4,22,23,24,25]. Whilst remote sensing offers many advantages, there remains a clear need for on-site data collection to validate and improve models [26,27,28,29,30]. Manual data collection is both hazardous and expensive, with variations in measuring techniques introducing the risk of errors and conflicting evidence [31,32]. We have designed, built and deployed a wireless sensor network (WSN) to address the limitations of existing peatland monitoring techniques, identify potential compromises and issues, and inform future developments in the field.

## 2. Materials and Methods

**Figure 1:**
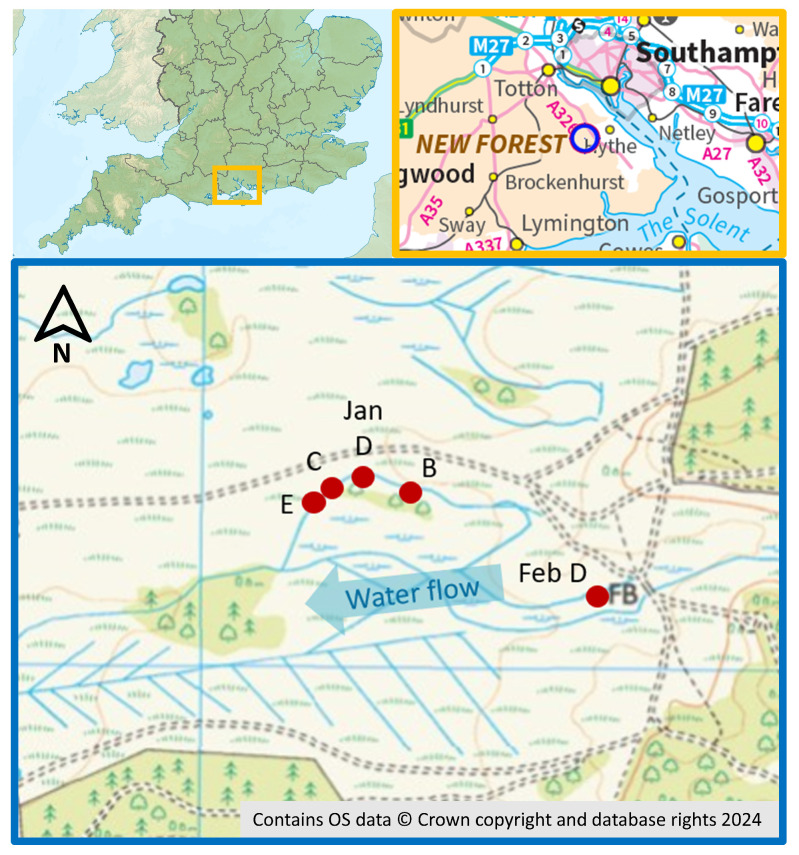
Maps showing the location of the sensor node deployment. (**Top left**): location within England; (**Top Right**): regional site location; (**Bottom**): node locations within Dibden Bottom (Ordnance Survey Map Reference: SU3906).

This research reports on a wireless sensor network comprising four sensor nodes and a gateway, which was deployed in a New Forest peat site in the UK for 2 months (Figure 1).

### 2.1. System Objectives

Our hypothesis is that a wireless sensor network can reduce the cost of peatland monitoring and provide higher sampling frequencies than current manual data collection techniques. The type of system that would support this hypothesis would need to meet four essential objectives:

1. Collect useful and meaningful data for monitoring peatland health.
2. Be compatible with the peatland environment.
3. Be easy for those unfamiliar with IoT to use.
4. Reduce long-term peatland monitoring costs.

### 2.2. Design Requirements

Stemming from the system objectives above, the following requirements were identified for the wireless sensor network and were used to guide the design development:

- Each sensor node should be capable of detecting carbon dioxide, methane, air temperature, humidity, soil moisture and soil temperature levels.
- Each sensor node should take measurements at least hourly.
- The system must be able to operate in the conditions expected for the site—i.e., wet ground, rain, frost−10 to 35 °C, and variable sunlight.
- The system may not rely on any external power supply, such as mains electricity.
- The system should minimise the risk of disturbance or harm to wildlife.
- The system should operate without human interaction for a minimum of 1 month.
- Collected data should be transmitted wirelessly and at least daily.
- The system should be portable and easy to deploy and retrieve.
- The system cost should be comparable to or cheaper than a similar month-long manual study.

### 2.3. System Architecture

**Figure 2:**
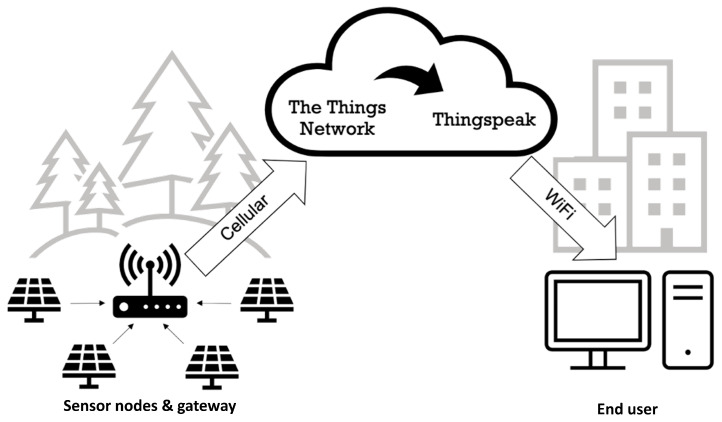
The overall architecture of the wireless sensor network. The local network of nodes (**left**) collects and transmits data to a gateway node, which forwards data to The Things Network via cellular networks. In the Cloud (**centre**), these data are again forwarded on to Thingspeak. Data on Thingspeak can then be viewed on a computer using WiFi (**right**).

The architecture of the sensor network is shown in Figure 2. Sensor nodes in the field collect and transmit data over LoRaWAN to the gateway device. Data from the gateway are uploaded to The Things Network, using a cellular connection. ThingSpeak reads data from The Things Network and formats it into graphs for immediate viewing online. The data can also be downloaded from Thingspeak to a computer for further processing.

LoRaWAN was selected as the wireless networking protocol for local communication between sensor nodes and the gateway, as this protocol is low-power, long-range and supports a high network capacity [33]. Low-cost LoRa-compatible devices are also widely available on the market. Sigfox is the only comparable wireless network, in terms of range and power consumption, but network coverage is managed by the Sigfox company (Labège, France) and remains incomplete in remote regions [33,34].

As preexisting LoRaWAN coverage is likely to be insufficient in remote areas, a local gateway node is also implemented. This receives data packages from the sensor nodes and transfers them to cloud services using cellular data, which offers better coverage.

The Things Network (TTN) [35] and Thingspeak [36] are two popular IoT web services, that handle data forwarding, decoding and storage. Both websites allow the download of sensor data to other devices. Many alternative cloud-based IoT services exist; TTN and Thingspeak were selected as they are reliable, free and accommodate most IoT devices. Furthermore, their data handling processes are transparent and easily customised by the user.

### 2.4. Gateway Node

The gateway node is based around a Milesight IoT UG65 Semi-Industrial LoRaWAN Gateway. This gateway was selected for its low operating power consumption (2.5 W), flexible input voltage and tolerance for dust, moisture and temperature variations [37]. The gateway is powered by two 30 W solar panels, which charge a 12 V 10 Ah LiFePO_4_ battery through an EPEVER LS1024E PWM solar charge controller (Beijing Epsolar Technology Co., Ltd., Beijing, China) [38].

The estimated daily energy generation from this system, *E*, is as follows:

(1) $$E=W\cdot H_{S}\cdot \eta ,$$

where *W* is the power rating of the solar panels, $H_{S}$ is the daily peak sun hours, and η is the system efficiency.

Using *W* = 60 W, $H_{S}$ = 2.9 h [39], and η = 0.75, this gives an estimated daily power generation of 130.5 Wh.

The estimated daily power consumption of the gateway was 30 Wh, giving a predicted safety factor of 2.175. A fully charged battery should power the gateway for up to 2 days without sunlight.

### 2.5. Sensor Node

**Figure 3:**
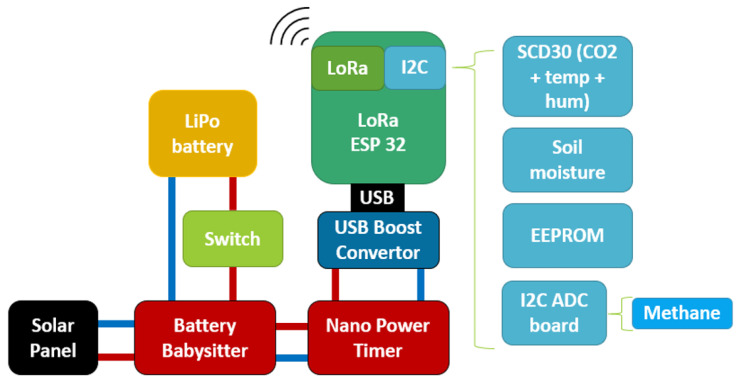
The architecture of the sensor nodes.

The overall design of the sensor nodes is shown in Figure 3. The Heltec ESP32 WiFi LoRa 32 (V2) microcontroller (Chengdu Heltec Automation Technology Co., Ltd., Chengdu, China) was selected for the nodes, as it is low-cost, has built-in LoRa capabilities and LiPo battery management, and supports a low-power sleep mode [40]. The module is programmed over USB using the Arduino development environment. An electrically erasable programmable read-only memory (EEPROM) module is incorporated to back up data in the case of poor signal or gateway failure.

A 3 W 5 V solar panel is fitted to each sensor node. The node power storage is a rechargeable 10,000 mAh 3.7 V Lithium-Polymer battery. The daily energy generation is calculated again using Equation (1). Using *W* = 3 W, $H_{S}$ = 2.9 h, and η = 0.5, this gives an estimated daily power generation of 4.35 Wh. A lower value of η is used to account for the horizontal inclination of the solar panels on the sensor nodes.

A daily consumption of around 3 Wh was measured for the sensor nodes. This safety factor is lower (1.45) than that for the gateway but a fully charged battery should support a sensor node for 12 days without sunlight.

#### 2.5.1. Sensor Selection

Three sensor modules are used within each node: a Sensirion SCD30 [41], an Adafruit STEMMA capacitive soil moisture sensor [42] and an NGM2611-E13 methane sensor [43].

The SCD30 uses nondispersive infrared technology to measure CO_2_ levels from 0 to 40,000 ppm, with an accuracy of ±30 ppm + 3% in the 400–10,000 ppm range. The module also measures relative humidity from 0 to 100% (±3%) and air temperature from −40 to 70 °C (±1 °C in the 0–50 °C range) [41].

The soil moisture sensor outputs unitless values ranging from 200 to 1015 with a higher value representing a higher soil moisture content. The sensor must be calibrated in the intended soil, as soil capacitance is affected by soil mineral content and porosity. On this occasion, the site permission did not allow the extraction of soil for calibration purposes, so for the duration of this study, the sensors were used in an uncalibrated state to monitor relative variations in soil moisture content. The soil moisture sensor also contains a low-precision temperature sensor within the ATSAMD10D14 chip. This temperature sensor has a raw accuracy of ±10 °C but with the refinement applied in the Adafruit software (Version 1.4.1) ±2 °C is typical [42].

The NGM2611-E13 is a module designed for use in natural gas leak detectors. The sensing element in the module is a TGS 2611-E00. The sensitivity characteristics plot provided in the Figaro TGS 2611 datasheet covers methane concentrations from 300 to 10,000 ppm [43]. As such, further characterisation of the sensor response at lower methane concentrations was required.

#### 2.5.2. Sensor Calibration

**Figure 4:**
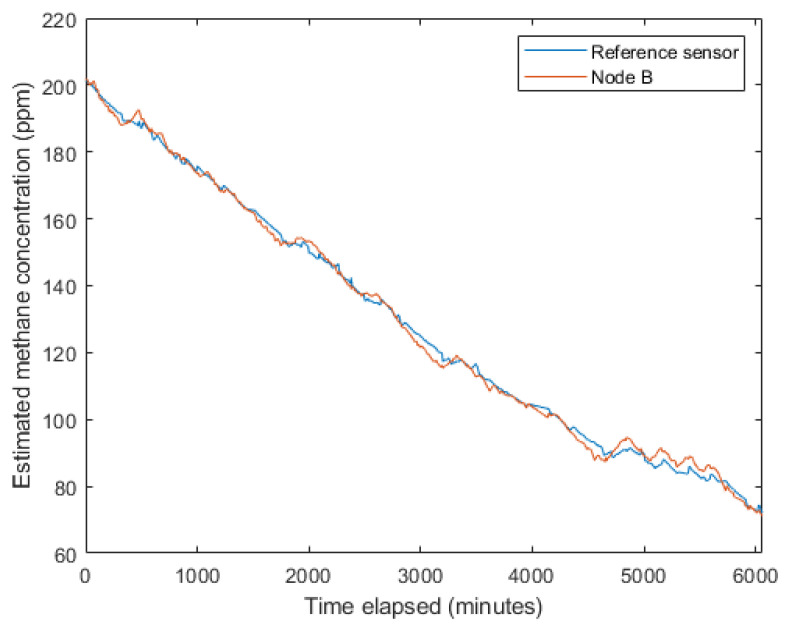
Estimated methane concentration from the trained models for the reference sensor and the Node B field sensor during the pre-deployment calibration experiment.

The sensor nodes were co-located in a vacuum for 4 days with a previously calibrated reference sensor. This process is explained in detail in [44]; 200 ppm methane in air calibration gas was injected into the chamber, then the vacuum chamber inlet was opened to allow the methane levels in the chamber to decrease gradually (Figure 4). During the experiment, the relative humidity ranged from 60% to 63%, the temperature ranged from 19.3 °C to 24.6 °C and the estimated methane concentration from 200 ppm to 72 ppm.

A model of the same form as was employed on the reference sensor was then trained on the collected data to calibrate the field sensors (Figure 4).

**Figure 5:**
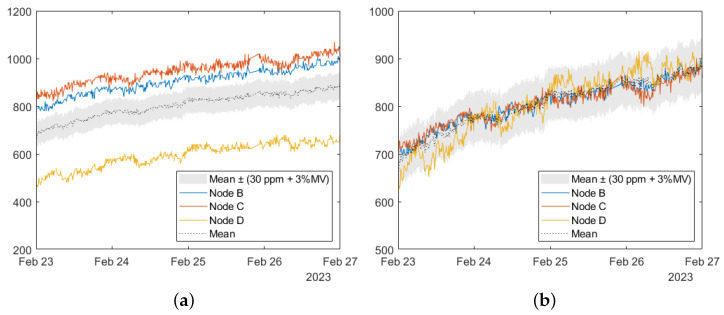
CO_2_ sensor outputs from the pre-deployment calibration experiment. (**a**) Raw sensor outputs (**b**) Calibrated using relative scaling factors. The manufacturer’s stated deviation, 30 ppm + 3% of the measured value is indicated by the shaded region.

The CO_2_ sensors were calibrated relative to one another during the same experiment as the methane sensors. The sensor nodes were co-located in a vacuum chamber for 4 days and the CO_2_ sensor outputs were recorded (Figure 5a). The mean from the sensors was calculated and a scaling factor relative to this mean was applied to the output from each sensor. This brought the variability between sensors to within the manufacturer’s stated deviation, 30 ppm + 3% of the measured value (Figure 5b).

The soil moisture sensor readings were converted to approximate saturation by mapping the maximum sensor output to 100% saturation and the sensor output in air to 0%.

#### 2.5.3. Node Operations

Every 30 min, the power timer turns on the microcontroller and sensors. Readings are taken from each sensor; in this process, 10 readings are taken over a duration of a few seconds and the average value is retained. The sensor readings are encoded as described in Section 2.5.4, and saved to the EEPROM data storage. The sensor node then attempts to connect to The Things Network using Over The Air Activation (OTAA) in order to transmit the data. Following this, the power timer is reset and the node is returned to sleep mode.

Following the first deployment period (December 2021–January 2022), a timeout feature was implemented, which activated the sleep procedure after 2 min, regardless of whether the sensor node had successfully connected to The Things Network. This prevented excessive power consumption when the gateway was offline.

#### 2.5.4. Data Format

**Figure 6:**
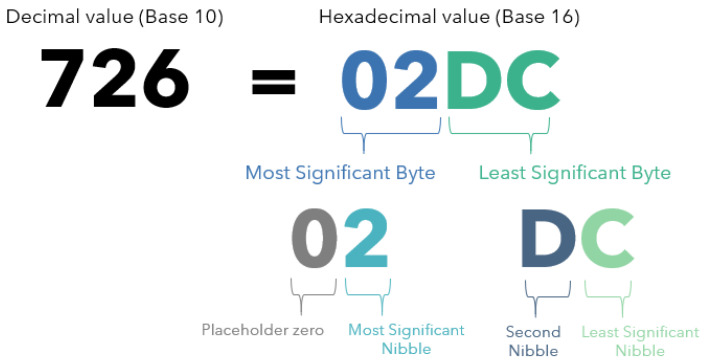
A diagram showing the components of the standard hexadecimal encoding format.

To reduce airtime and power consumption, a novel datastring encoder was devised. Contrary to the default approach of encoding data as hexadecimal bytes, this encoder uses nibbles—i.e., individual hexadecimal digits. For example, in a standard system, if the maximum value reported by a sensor is 726, in hexadecimal notation, this would usually be encoded across 2 bytes as “02DC”. The issue with this approach is that the first digit is never used but is always transmitted as “0” (Figure 6). The system described below removes this issue, thereby reducing transmission length.

The sensor data are encoded as follows, with an example shown in Table 1:

1. Retrieve the value from a sensor (Reading).
2. Round the data to the accuracy of the sensor (Rounded).
3. Subtract the minimum possible reading of the sensor from the current value (Vs min).
4. Scale up the current value to an integer (Decimal to send).
5. Convert the value to hexadecimal nibbles (Hex to send).
6. Concatenate the resulting hexadecimal values into a single string for submission.

Upon reception, the string is then spliced, bit-shifted and decoded by reversing the process above to retrieve the original values. The receiver requires only the order of the variables sent, the accuracy and the minimum value of each sensor; the easiest approach is to standardise this format before implementation.

#### 2.5.5. Error Filtering

In any sensing system, there is a risk of errors being generated at the point of sensing, during pre-processing and during transmission or storage. This wireless sensor network is no exception, so a process was developed to identify and remove errors in the data. If any of the following criteria were met, the sensor reading was rejected as spurious:

- Humidity exceeding 100%
- Temperature exceeding 100 °C
- Temperature below −30 °C
- Battery charge value exceeding 100%
- Extreme outliers for CO_2_ (outside the range 100 to 3000 ppm)
- Extreme outliers for methane (raw sensor voltage outside the range from 0.4 to 1.6 V)
- Extreme outliers for soil moisture (outside the raw sensor data range of 200 to 1200)

### 2.6. Site Selection

The selected installation site was Dibden Bottom, a valley mire located within the New Forest National Park (Figure 1). Within the site, water flows from upstream in the East, across the peat bog and down to the West.

**Figure 7:**
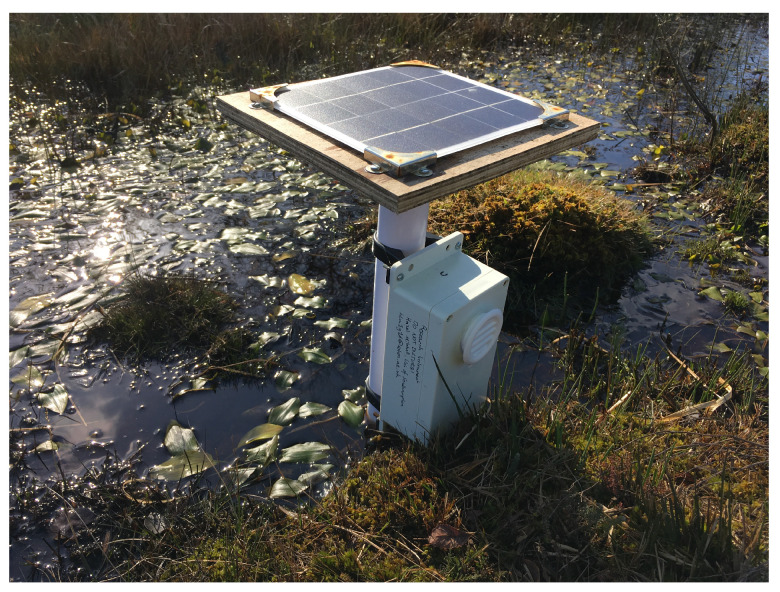
A photograph of Node C following deployment.

Figure 7 shows Node C in position at the site as an example of sensor node installation; the vertical PVC tube is inserted into the soil such that the soil moisture sensor is submerged to the correct depth and the air inlet is not blocked.

For the December 2021–January 2022 period, sensor node D was located within range of the gateway (labelled “Jan D” in Figure 1). To test the offline functionality, for the February 2022 period, the node operated in offline mode in the new Easternmost location (labelled “Feb D” in Figure 1), recording data on the local EEPROM rather than transmitting to the gateway.

## 3. Results

### 3.1. Overall System Specifications

Table 2 summarises the properties and capabilities of the presented wireless sensor network.

### 3.2. Recorded Data

**Figure 8:**
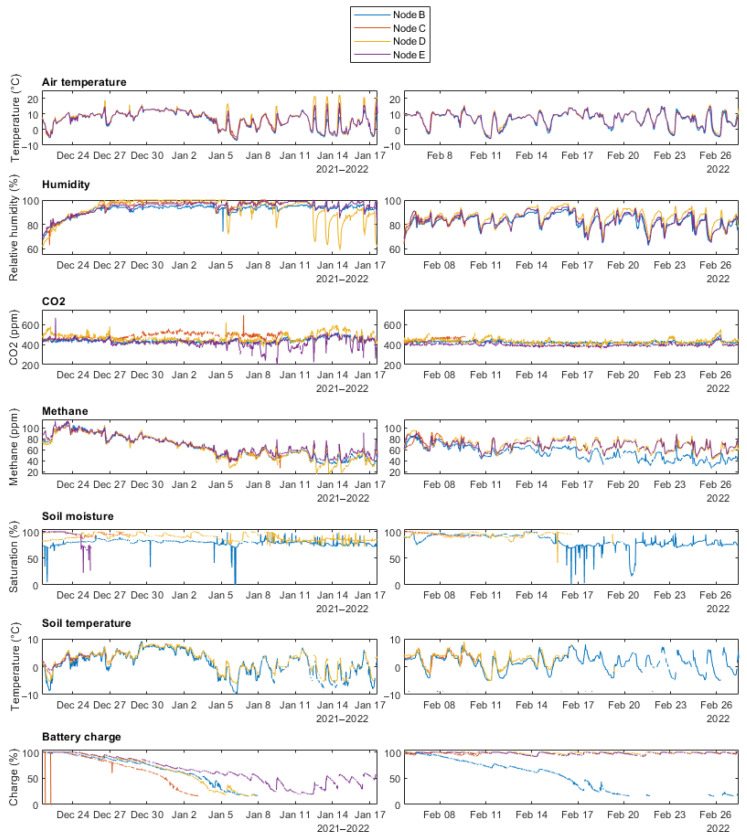
Overview of the data collected by the sensor nodes across the two deployment periods, following error filtering.

A total of 7797 data points were collected across five locations from 21 December 2021 to 27 February 2022. Figure 8 shows the time series plots for the two study periods (21 December 2021–18 January 2022 and 5 February–27 February 2022).

Similar trends were observed for temperature and humidity across all nodes. More variability between nodes was observed in the CO_2_ and methane data but the overall trend was similar across nodes. CO_2_ levels of around 400 ppm were observed for the majority of both study periods, with some short-scale variation. Methane levels were highest at the start of the first deployment, in December, tapering off towards the start of January and then remaining more stable through January and February.

Where present, soil temperature follows a similar trend to air temperature for all nodes. Some data are missing from the soil moisture plots because, at different points in both study periods, these sensors failed (see Section 3.3.2). The increased sunlight in February, compared to the earlier December–January study period, can be inferred from the higher mean battery charge (88% charge in December–January vs. 66.6% charge in February).

### 3.3. System Performance

As this research reports in field deployment, we include in this section some practical experience and reflections on how the system and its components performed in a real-world scenario.

#### 3.3.1. Gateway Performance

Two limitations were encountered with the gateway node: limited wireless range and insufficient power generation. The first was primarily due to the topology of the site. There were multiple trees surrounding the gateway and the terrain was uneven, sloping uphill at the East end of the site. As such, the LoRa communication range of the gateway was less than 500 m. This would likely be improved if the gateway was placed at a higher altitude, with a clear line-of-sight to the sensor nodes. The usable duration of the gateway was much shorter than expected. The battery charge dropped below the threshold needed to power the gateway 3 days from the start of the first deployment. The battery was replaced with a fully-charged spare on 11 January, which supported the gateway until the end of the first deployment but with more frequent offline periods from 14 January onwards. Similarly, in the second deployment, the gateway remained online for the first 3 days, and then became unreliable. The battery was replaced again with a smaller spare on 11 February and the gateway went offline again on 12 February. Multiple factors contributed to this issue. Despite mild temperatures, December 2021 was one of the darkest months in the past century in England, with only 30.6 h of sunshine across the whole month [45]. The solar panels were also slightly shaded by branches. This was compounded by the solar charge controller, which did not charge the batteries if the input from the solar panels was too low and disabled the output to the gateway if the battery voltage dipped below a safety threshold.

#### 3.3.2. Sensor Issues

The following issues were identified during the field deployment of the sensor network:

- Null values—created when a sensor fails to return a reading. The recorded value will be either zero or the minimum value the sensor can return;
- Saturated values—often caused by sensor faults or disconnection, leading to the maximum possible sensor output being recorded;
- Random values—multiple potential causes, often difficult to diagnose or prevent but usually short-lived and easy to filter out as they often affect multiple sensors in a given node at the same time. Causes may include moisture ingress, unstable wireless communication, or malfunctioning components;
- EEPROM data corruption—errors created when writing to the EEPROM. This issue occurred most commonly when the battery charge fell below 16% and was caused by a bitwise shift in the stored data. This was reversed in post-processing to repair the data.

**Figure 9:**
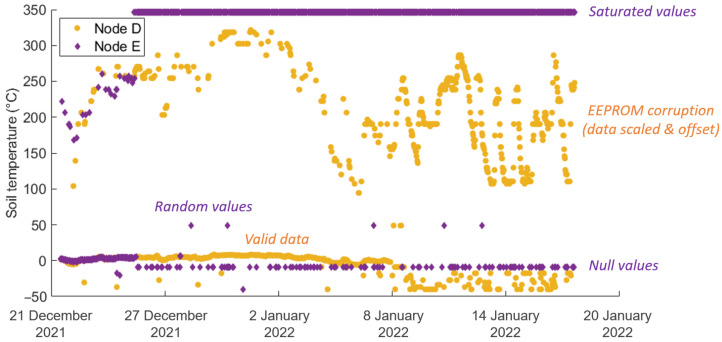
December 2021–January 2022 raw soil temperature data from nodes D & E, annotated to highlight examples of each error type described in Section 3.3.2.

Figure 9 shows examples of each of these types of errors seen in the raw soil temperature data extracted from the EEPROM data storage.

Taking Node D as an example: in the first deployment, 6% of the CO_2_ measurements, 6% of the methane measurements, 30% of the battery charge, 11% of the soil moisture and 9% of the soil temperature measurements retrieved from the EEPROM were filtered from the data as likely errors. No errors were found in the air temperature and humidity data. Similar proportions were observed across the other sensor nodes in both deployments. Samples with unrealistic CO_2_ concentration values were consistently accompanied by errors in the methane, soil moisture and temperature, and battery charge readings. However, in the corresponding data received by ThingSpeak at the same timestamp, these errors are not present. For example, the 12 January 2022 20:44 sample recorded a CO_2_ concentration of 370 ppm on ThingSpeak, versus 5940 ppm on the EEPROM. This implies that the issue lies with the EEPROM module, which intermittently corrupted stored data.

The soil moisture and soil temperature sensors on Nodes C and E failed early in the December–January deployment (on 28 and 25 December, respectively), likely due to water ingress or sensor disconnection. This explanation is proposed because saturated and null values are observed after those dates (Figure 9). The lack of soil moisture and soil temperature data from Node E in the February deployment was attributed to a sensor wire becoming disconnected during transit to the site, as the wire was disconnected when examined upon retrieval and the recorded soil moisture value throughout the deployment was saturated at 4095.

#### 3.3.3. Limits on Deployment Duration

At the time of writing, the WSN has been tested in the field for a total of 50 days, with the longest continuous deployment lasting 28 days. During the first deployment, a software issue was discovered where if OTAA failed on a sensor node, the sleep procedure would not be activated, causing increased power consumption. As such, the battery charge levels of all four nodes declined over most of the deployment period, as seen in Figure 8. As expected, being located in the least shaded location, Node E retained the highest battery charge: 63%, at the end of the Dec–Jan deployment. Conversely, Node C was positioned in slight shade and its battery charge declined the most rapidly, falling to 16% by 3 January, four days earlier than Nodes B and D.

After this issue was addressed for the second deployment, Nodes D and E maintained battery charge levels above 90% for the entire deployment period (Figure 8). The cause of failure for Node C in the second deployment is most likely due to the 5 V wire between the sensor layer PCB and the control layer PCB coming loose. The Node functionality was restored following retrieval and reconnection of this wire. Node B showed a similar decline in battery charge across both deployments. This may have been due to a loose solar panel connection or the updated software may have not been correctly installed on the microcontroller. Setting aside these issues, the sensor nodes showed the potential to operate indefinitely from a power generation perspective. As such the current limiting factor for deployment duration is the capacity of the EEPROM data storage.

Using the compression approach outlined in Section 2.5.4, the datastring length for a standard transmission from the sensor node was reduced from 26 nibbles to 20 nibbles, a size reduction of 23%. This has the additional advantage of increasing the number of readings that can be stored on the EEPROM module. The Sparkfun EEPROM module used has a memory capacity of 64 kilo-bytes. The first four bytes are used to hold the memory location of the most recent datastring that was saved. The number of days of data that can be saved is, therefore, calculated by dividing the memory capacity by the number of daily readings and the size of each datastring. With no compression, and readings taken every 30 min, 102 days of data can be stored. Using the compression approach above, this is increased to 121 days.

This datastring compression can also reduce the power consumption of the sensor nodes by reducing the transmission duration. Table 3 shows an example power consumption estimation for the sensor nodes, configured without the custom datastring encoder and with the encoder reducing the datastring length and transmission duration by 23%. In this example, using the encoder would impart a corresponding reduction in power consumption of 4.5%.

## 4. Discussion

### 4.1. Performance against System Objectives

The following subsections assess how well the WSN met the research objectives and design criteria set out in Section 2.1 and Section 2.2.

#### 4.1.1. Objective 1: Useful and Meaningful Data

The SCD30 sensor performed well over both deployments, as did the NGM2611-E13 methane module. The soil temperature sensor is located at the surface of the soil, and the soil temperature measurements closely track the air temperature. Subsurface soil temperature measured with a more accurate sensor would be a more useful metric for future deployments.

The soil moisture sensors proved less useful, with a large fraction of the soil moisture data being lost in both deployments due to condensation buildup on the sensor PCB. Though not suitable for this site, an instrumented dip-well sensor might provide more meaningful data (i.e., water table depth) for subsequent studies.

On a similar note, as gases in the air near the sensor node are able to freely diffuse, the levels of a given gas measured by the sensor represent emissions from the surrounding area, rather than exclusively the point directly below the sensor node. This is advantageous in sites with varied micro-topographical features, such as pools, hummocks or ditches, or varying vegetation, which have been shown to impact greenhouse gas emissions [46].

Conversely, soil moisture is only measured from the soil in direct contact with the sensor. To further investigate potential correlations between site-wide emissions and soil moisture, without increasing the number of nodes that need to be deployed, the development of a wider area soil moisture sensor would be beneficial.

In keeping with the component selections, low-cost approaches were employed to calibrate the sensors. In the case of the methane sensors, this resulted in unrealistically high methane concentration estimations. This is attributed to the lower temperature (−7–22 °C vs. 19–25 °C), higher humidity (59–100% vs. 60–63%) and lower methane concentration experienced in the field compared to the calibration experiment conditions. At the time of writing, calibration of the methane sensors at low methane concentrations (<10 ppm) is ongoing. As such, the quantitative methane concentration estimates from this deployment cannot be treated as an accurate representation of the true concentration at the site. However, trends and short-term events can still be analysed from the presented methane data. This provides an advantage over manually collected data with coarse temporal resolution, from which trends and transient events are difficult to ascertain.

At the design conception stage, sunlight was not anticipated to be an important factor influencing methane and CO_2_ emissions. As such, light sensors were not included in the sensor nodes. Whilst battery charge rates offered a useful proxy for incident solar radiation in this study, conventional light sensors would provide direct measurements and would still function in summer, when the batteries would often be fully charged, preventing their use as a proxy for a light sensor.

Arguably, the greatest strength of the WSN is its high sampling rate. The sampling frequency of CO_2_ and methane levels using manual chamber methods varies greatly between existing studies. It is rare for such studies to take more than two readings per day. Some rely on as few as five measurements in a year [47]. It is evident from the data shown in Figure 8 that considerable variations in local conditions and greenhouse gas concentrations occur over short time periods, often less than an hour. Flux towers have been used in peatland studies to obtain data with a temporal resolution of 30 min [48,49]. The WSN presented here currently operates at the same rate but can easily be configured to increase the sampling rate to every 10 min. Adaptive data acquisition scheduling on sensor nodes could also be implemented. The current node solar panels are sufficient to support operation year-round when placed in full sun and would easily have the required capacity for more frequent measurements in summer. Reducing sampling frequency on cloudy winter days (which presented less variability in GHG concentration) and increasing sample rates when more power is available would optimise the functionality of the sensor network. This high temporal resolution can be crucial when seeking to understand the complex chemical and biological processes that influence peatland emissions.

#### 4.1.2. Objective 2: Compatible with the Peatland Environment

As mentioned above, the soil moisture sensors were found to be susceptible to water ingress and condensation. In future iterations, these sensors could be replaced with a more expensive but waterproof alternative. Otherwise, the sensor nodes performed well in the peat site, over a wide range of temperatures and weather conditions. Only sensor node E showed evidence of visible condensation within the enclosure upon its retrieval at the end of the second deployment but this did not appear to have affected the node’s functionality.

The size of the gateway solar panels was limited by transportation capabilities and few viable installation locations. The installation location needed to be secure and above the grazing height of local animals; as such, the only suitable option was chaining the gateway and panels to the lower branches of a sturdy tree. This meant that the panels were not positioned in full sunlight and the reduced sunlit duration through winter led to the gateway losing power within a week of deployment in both studies. Within the context of this study, this was not an issue, as all the data collected by nodes were also saved to the EEPROMs and downloaded upon retrieval of the nodes. However, longer future deployments may rely more heavily on gateway reliability, as EEPROM storage has a finite memory capacity.

To minimise mass, volume and cost, it would be beneficial to reduce the gateway power consumption through adaptive transmission scheduling, rather than increasing power generation through larger solar panels or additional sources like wind power. This could involve buffering messages within the sensor nodes and transmitting data less frequently during periods with lower power availability. Alternatively, the sensor nodes could be adapted to transmit data directly over 4G, instead of using a local gateway for data forwarding.

The sensor nodes were discreet and left no visible disturbances on the deployment site following retrieval. There was also no evidence of damage to the WSN by local wildlife.

#### 4.1.3. Objective 3: Ease of Use

The full WSN (four sensor nodes, gateway and solar panels) can be fitted and carried in a pair of 30 L rucksacks. Therefore, with two people, it is easy to carry everything needed for a full deployment on foot. Installation of the nodes is simple: the solar panel is mounted on top of the PVC tube and the tube is pushed vertically into the peat.

The option to view live data remotely is a convenient feature; site conditions can be assessed even when weather conditions would make manual data collection dangerous or impossible. Due to the aforementioned gateway issues, this feature was not available for the full duration of either deployment but the principle was successfully demonstrated. Even considering this issue, the local data storage on each node performed well, allowing the untransmitted data to be recovered when the WSN was retrieved. Once installed, provided power generation is sufficient, the WSN requires no human interaction to function throughout the deployment.

#### 4.1.4. Objective 4: Long-Term Cost Reduction

The material costs for gas enclosures are highly variable depending on the materials used—many researchers opt to construct DIY chambers which would likely be less expensive than the sensor nodes presented in this study. However, the main cost of monitoring using enclosure techniques is the recurring labour cost associated with travel to the site, manual sampling and lab analysis of gas samples. Excluding deployment and repairs, the wireless sensor network eliminates these costs.

Flux tower costs are a function of their height and instrumentation, but costs in the tens of thousands to hundreds of thousands (GBP) are typically cited [50,51,52] but they take measurements autonomously once installed.

A survey conducted by Natural England in 2011 found that estimated monitoring costs across 10 peatland projects ranged from £200 to £70,000 per year, with an average annual cost of £18,720 [6]. Of the projects surveyed, only two reported monitoring greenhouse gas emissions, both on a monthly basis.

The total material cost of the presented wireless sensor network (four sensor nodes + one gateway + one month of SIM fees) was £1350. Following setup, the only recurring costs are data transmission fees and repairs. This represents less than 10% of the estimated average annual cost of current monitoring approaches.

It should be noted that monitoring costs vary greatly depending on the site scale, location, monitored parameters and monitoring frequency. The costs cited in the Natural England survey include additional parameters not measured by the WSN, such as vegetation cover and biodiversity. However, the highest monitoring costs were associated with carbon storage (£43,000, from one response), remote sensing (£10,000, from one response) and hydrology function (£5583.33 average from five responses) [6]. Each of these monitoring categories overlaps with the capabilities of the WSN, suggesting that even partially substituting existing techniques with a WSN has the potential to reduce costs drastically.

### 4.2. Datastring Encoder

The proposed datastring encoder is designed with the specific limitations of environmental monitoring using a WSN in mind, as other well-known approaches are unlikely to confer the same benefits. Huffman coding is more suitable for scenarios in which a wide variety of characters may need to be transmitted, i.e., text messages. With hexadecimal numerical data, only 16 characters are used. It is also difficult to predict the relative probabilities of these characters in field data before deployment. Run-length encoding could benefit monitors measuring stable conditions or categorical data but repeated measurements are relatively unlikely in environmental monitoring. In use cases like this, where data are transmitted in real time, rather than in batches, the potential size reduction in run-length encoding is further reduced.

Table 3 presents an example in which the datastring length is the main factor controlling the duration of transmissions. In reality, other factors can affect the duration, such as Over-The-Air activation protocols. The benefit of compressing the transmitted data will be greater for larger data packages. Ultimately, the decision to employ this type of data compression should be made according to the use case, taking into account the size of collected data, transmission frequency, consequences of data loss, and power efficiency of the transmitter.

### 4.3. Limitations and Future Work

The sensor nodes used in the WSN do not take into account the directionality of fluxes—where a flux tower uses a 3D anemometer to discern whether emissions are leaving the site or simply moving within a site, the sensor nodes measure only the ambient concentration of gases. This may exacerbate certain aerodynamic effects, such as gases being trapped in a boundary layer of vegetation. The gas sampling ”footprint” of the sensor nodes is also uncertain; future investigators may seek to quantify this through modelling and controlled field experiments.

Mobile data coverage remains insufficient at some locations and can be further impeded by precipitation. Future iterations should investigate alternative backhaul options to mitigate this issue. This may be addressed by reconfiguring the sensor network itself, by employing a multi-hop or mesh network to expand coverage. Larger deployments may consider establishing a more permanent LoRaWAN gateway. Alternatively, satellite non-terrestrial network communications could be enabled as a backup data upload route.

The UG65 LoRaWAN gateway used supports up to eight end devices simultaneously transmitting data, and up to 2000 total end devices on the network. As such, the system is easily scalable for larger deployments without purchasing additional costly gateways. With larger deployments, it may be necessary to schedule node transmissions to prevent clashes between transmissions from the nodes.

The modular design of the wireless sensor network makes it possible to reconfigure the system for alternative applications. For example, the temperature and humidity sensors could be replaced with pH, turbidity and water pressure sensors, to facilitate hydrological monitoring. Alternatively, with the addition of particulate matter sensors, the wireless sensor network could be utilised as an early detection system for wildfires. With alterations to the enclosure and mounting system, the WSN could also be deployed to monitor greenhouse gas emissions from other land types, such as salt marshes, lakes, agricultural land and woodlands. As appropriate sensors enter the market, including other relevant gases (nitrogen dioxide, carbon monoxide and volatile organic compounds) would help to construct a more comprehensive picture of the contribution of these sites to global emissions.

## 5. Conclusions

The development and deployment of the described IoT sensor network enabled real-time monitoring of a peatland site, with improved temporal and spatial resolution, compared to traditional manual data collection. The wireless sensor network recorded mesotope-scale measurements on a half-hourly basis but can be reconfigured to higher sampling frequencies. A total of 7797 data points were collected by the network’s four sensor nodes over the 2-month deployment period.

Though optimised for peatland deployments, the development of this wireless sensor network revealed several considerations that are relevant to other applications. Examples include compressing data based on sensor reliability, rather than precision and using local averaging to minimise the influence of minor sensor malfunctions. The novel datastring encoder proposed in this paper reduced the transmitted datastring by 23%, which is beneficial for reducing storage and power consumption.

Future work should investigate more resilient backhaul approaches, such as mesh networks or direct communication from the sensor nodes to a mobile data network. Direct comparison with flux tower measurements and quantification of the sampling range of each sensor node is also recommended.

With further refinement, the presented sensor network shows potential as a new, low-cost means of carrying out detailed studies of peatland emissions and associated climate variables, with minimal human intervention and site disturbance.

## Figures and Tables

**Table 1:** Example of data encoding using SCD30 temperature data. The sensor has an accuracy of ±0.15 °C but provides data at a precision of 0.01 °C; therefore, these data are rounded to the nearest 0.1 °C.

	Reading	Rounded	Vs min	Decimal to Send	Hex to Send
Min. temperature	−40	−40	0	0	0
Max. temperature	70	70	110	1100	44C
Example temperature	32.62	32.6	72.6	726	2D6

**Table 2:** Top-level specifications of the wireless sensor network.

Parameter	Value
Sensor node mass	1 kg
Gateway node mass	5.6 kg
Total system mass	9.6 kg
Materials cost	£1350 ^1^
Monthly running costs	£8 ^1^
Sampling interval	30 min
Maximum theoretical deployment	121 days ^2^
Tested continuous deployment	28 days
Measured variables	Air temperature, relative humidity, CO_2_ concentration, methane concentration, soil moisture, surface soil temperature, battery charge level

^1^ Prices correct at time of writing. ^2^ Currently limited by EEPROM data storage capacity.

**Table 3:** Estimated sensor node power consumption calculations, comparing scenarios with and without compressing the data before transmission. Based on specifications from [40].

	Without Encoder	With Encoder	Units
System voltage	5		V
Measurements per day	48		
Measurement current	0.5		A
Measurement power	2.5		W
Measurement duration	60		s
Total measurement duration	2880		s/day
Total measurement energy	2		Wh/day
Transmit current	0.13		A
Transmit power	0.65		W
Transmit duration	60	46.2	s
Total transmit duration	2880	2217.6	s/day
Total transmit energy	0.52	0.4004	Wh/day
Sleep current	0.0008		A
Sleep power	0.004		W
Total sleep duration	80640	81302.4	s/day
Total sleep energy	0.0896	0.090336	Wh/day
Total energy	2.6096	2.490736	Wh/day

## Data Availability

The original data presented in the study are openly available at: https://doi.org/10.5281/zenodo.12759490.

## Abbreviations

The following abbreviations are used in this manuscript:

CH_4_	Methane
CO_2_	Carbon Dioxide
GHG	Greenhouse Gas
PPM	Parts Per Million
RH	Relative Humidity
RMSE	Root Mean Squared Error
SD	Secure Digital
WSN	Wireless Sensor Network
